# High-efficiency cellulose acetate/GO/CaCO_3_ for solar photodegradation of methylene blue

**DOI:** 10.1038/s41598-026-42390-6

**Published:** 2026-04-01

**Authors:** Sawsan Dacrory, Samir Kamel

**Affiliations:** Cellulose and Paper Department, National Research Centre, Dokki, Giza, 12622 Egypt

**Keywords:** Cellulose acetate, Solar photodegradation, GO, Methylene blue, Chemistry, Environmental sciences, Materials science, Nanoscience and technology

## Abstract

A solar photodegradation film for methylene blue (MB) removal has been fabricated using cellulose triacetate (CTA), graphene oxide (GO), and CaCO_3_. First, GO was prepared from graphite using the modified Hummers method, and its structure was confirmed by X-ray diffraction and Raman spectroscopy. CTA/GO film loaded with CaCO_3_ (CTA/CaCO_3_/GO) was prepared by a simple casting method, and its structure was characterized using Fourier transform infrared (FTIR) spectroscopy, X-ray diffraction (XRD), and scanning electron microscopy (SEM)/ energy dispersive electron spectroscopy (EDX). Hydrophilicity was investigated using contact angle measurements, which confirmed the effectiveness of GO as a surface-modifying agent, transforming the CTA/CaCO_3_ film into a more hydrophilic film. The solar photodegradation of MB dye by a CTA/CaCO_3_/GO film was investigated under varying pH, sorbent dosage, and contact time. The results showed that more than 90% of the MB dye was removed by CTA/CaCO_3_/GO under solar radiation after 2 h. The rate of solar photodegradation of MB dye by the CTA/CaCO_3_/GO film followed pseudo-second-order kinetics.

## Introduction

Water is essential for all living things on Earth to survive. Water scarcity has adversely affected numerous human achievements, including urbanization, agriculture, industrialization and animal production^1,2^. Wastewater containing dyes can be a significant environmental pollutant, affecting human well-being. One source of this dye contamination is the textile industry, which generates large amounts of colored wastewater^3^. Moreover, about 15% of the industry-used dyes are released into the environment after production and processing. Methylene blue (MB) dye, methyl orange, reactive black, rhodamine B, and Congo red are classified into cationic, neutral, and anionic dyes^4,5^. However, these dyes are non-degradable due to their chemical intricacy, specifically, MB dye, which is a synthetic heterocyclic aromatic (3,7-bis(dimethylamino) phenothiazine chloride tetra methylthionine chloride), and a cationic chemical compound^6^. MB is used as a colorant in paper, wool, cosmetics, silk, temporary hair colorants, textiles, cotton, food, and the pharmaceutical industry. Also, MB dye is known for its cardio-protective, antioxidant, antidepressant, and antimalarial properties^7^. There are many techniques for mitigating health hazards and environmental issues associated with dyes in water, such as adsorption, chemical oxidation, and phytoremediation^3^. In general, dye removal from wastewater has been thoroughly studied using conventional wastewater treatment processes comprising preliminary, primary, secondary, and tertiary stages. These treatment methods are inefficient in remediating MB dye from industrial wastewater^8^. Hence, advanced techniques are attracting the researcher’s attention to overcome the shortcomings of conventional approaches. These advanced techniques include reverse osmosis, membrane separation, electrocoagulation, chemical precipitation, oxidation process, and electrodialysis^9^. Unfortunately, these technologies have specific limitations, such as high energy, high running costs, chemical consumption, high capital investment, and substantial capital inputs^10^. In this respect, the photodegradation system is considered an inexpensive and straightforward approach to water purification. In addition, to save the environment, the photodegradation process should follow the green synthesis protocol^11,12^. On the other hand, owing to the chemical and physical properties of carbon-based materials, they have demonstrated significant potential for various environmental applications. Despite carbon’s low catalytic activity in photocatalytic reactions, carbon-based materials with diverse morphologies can still exhibit catalytic activity. Carbon-based photocatalysis has attracted considerable interest because carbon atoms can assemble into various structures and dimensions. Therefore, carbon-based materials are widely used in photocatalytic water purification^13^. Graphene derivatives, including graphene, graphene oxide and reduced graphene oxide have emerged as promising materials for wastewater treatment owing to their favorable properties^14^. In addition, the porous structures and large surface areas enable rapid mass transfer and expose more catalytic sites, thereby accelerating the catalytic reaction. To date, numerous studies have focused on the development of graphene-based heterojunctions with other semiconductors, such as TiO_2_, for the removal of methylene blue dye. As reported, the impurity bands of the carbon p orbitals caused the composite to exhibit visible light photocatalytic activity for the degradation of methylene blue^15^. So, the use of graphene oxide (GO) as a carbon-based material is emerging in wastewater treatment research; these materials have been shown to improve mechanical properties and impart robustness to polymeric scaffolds^16^. Previously, several studies have shown that incorporating GO into membranes enhances the water permeation, antimicrobial, and antifouling properties for reverse osmosis, ultrafiltration, and forward osmosis applications^17^. Due to its low cost, renewability, superior hierarchical porous structure, pH sensitivity, and abundant variety, calcium carbonate (CaCO_3_) is suitable for various environment-related applications^18^. It can be used as a modifier or combined with semiconductor photocatalysts with smaller band gaps than those of traditional semiconductors^19^. Polymeric membranes are essential in the field of water treatment, but their separation efficiencies are still inferior compared with adsorbents of high porosity, like activated carbon^20^. Moreover, the polymer nature used in membrane preparation remains a significant challenge.

Cellulose is the most abundant polymer on Earth^21,22^. However, because cellulose is insoluble in most solvent mixtures, including those typically used in membrane processes, cellulose derivatives are preferred in membrane processes. The most representative cellulose derivatives in the membranes are cellulose acetate, nitrocellulose, carboxypropyl cellulose, and carboxymethyl cellulose^23,24^. Among these derivatives, cellulose acetate is the preferred derivative due to its broad solubility in polar aprotic solvents and its suitability for acetyl group functionalization^25,26^. According to the above survey, in this work, a cellulose acetate film loaded with GO and CaCO_3_ was fabricated by a simple casting method. The incorporation of CaCO_3_ into the composite can enhance photocatalytic activity by promoting the uniform distribution of the other component on the surface. This, in turn, facilitates the adsorption and catalytic degradation performance of pollutants on the surface of the composite^27^. The prepared film was characterized using FTIR-ATR, XRD, and SEM/EDX. Moreover, the CA/ CaCO_3_/GO film was investigated for the photocatalytic degradation of MB in aqueous solution under sunlight.

## Materials and methods

### Materials

Graphite powder was acquired from Fisher Chemical. Cellulose triacetate (CTA) was purchased from Fluke Biochemical Co., Schaffhausen, Switzerland (M. wt. 37,000 g/mol, CAS number: 9004-35-7, purity = 40% acetyl groups). Potassium permanganate (KMnO_4_) and hydrogen peroxide (H_2_O_2_) were analytical reagents purchased from Alfa Aesar. Methylene blue (MB, 99%) (C_16_H_18_ClN_3_S·2H_2_O) was purchased from Rankem. All materials were used as received.

### Experimental

#### Preparation of GO

Graphene oxide (GO) was prepared using the modified Hummers method, as described in our previous work^28^. In a three-neck round-bottom flask, 3 g of graphite and 18 g of KMnO4 were placed, and a mixture of phosphoric acid and sulfuric acid (360:40 mL) was added and agitated in an oil bath. After 2 h, the mixture was cooled to room temperature in an ice bath, and H_2_O_2_ (3 mL, 30%) and H_2_O (400 mL) were added dropwise. The suspension was then centrifuged at 5000 rpm for 10 min. The residue was repeatedly rinsed with water, ethanol, 30% HCl, and double-distilled water until the pH was neutral. The solid was collected by centrifugation and then dried in a vacuum oven at 85 °C. Raman spectroscopy was used to confirm graphite oxidation; it employs visible- or near-infrared laser excitation to analyze chemical bonds. Raman spectra were collected using a WITEC Focus Innovations Alpha-300 microscope with an excitation wavelength of 785 nm.

#### Film preparation

**Scheme 1:**
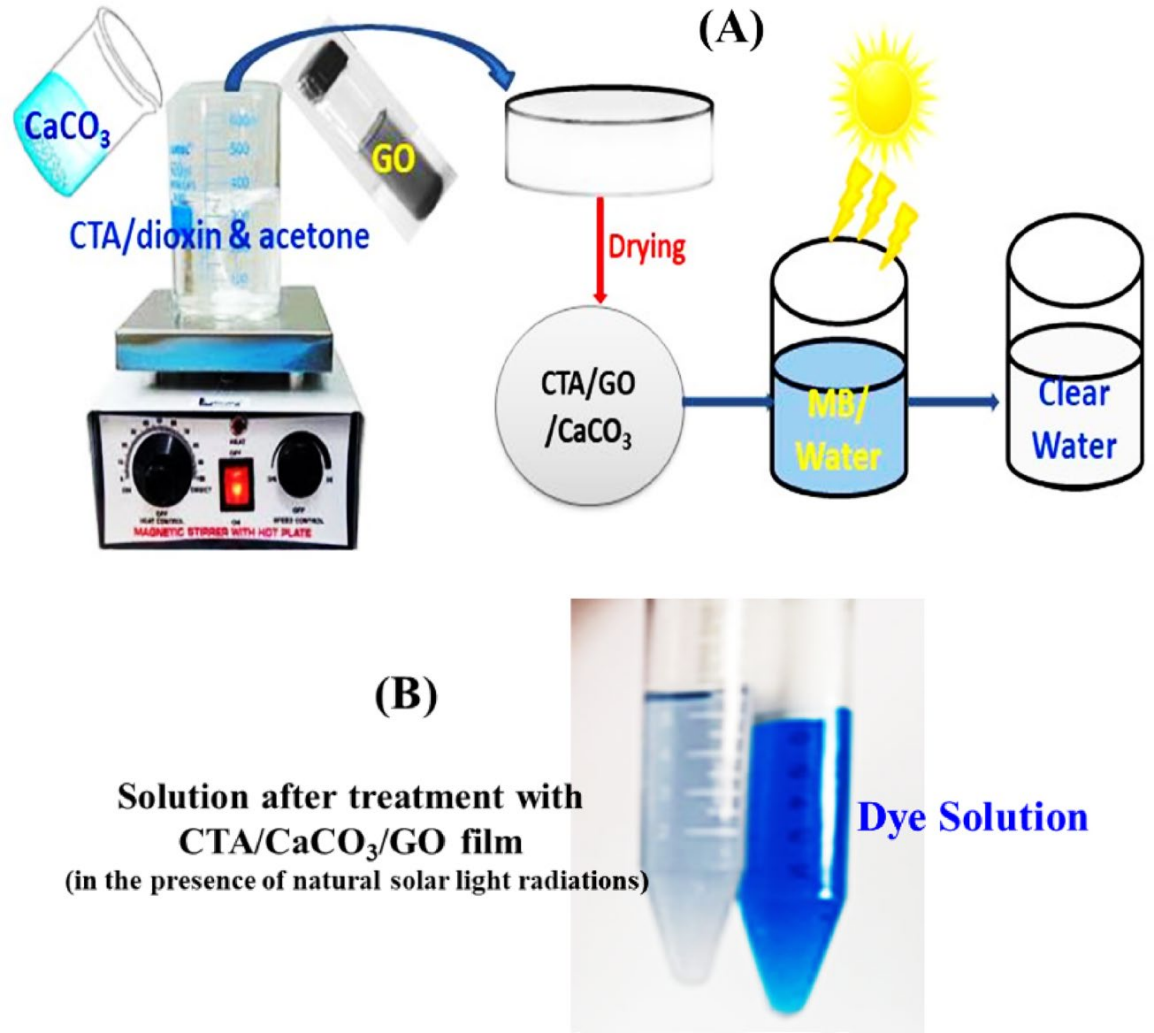
Schematic preparation of CTA/CaCO_3_/GO film (**A**), and its application in dye removal (**B**).

1 g CTA was dissolved in 50 mL of dioxin/acetone mixture (1:1) by magnetic stirring overnight at room temperature. After complete dissolution, the ultrasonicated acetone-dispersed GO (0.1 g) and CaCO_3_ (0.1 g) were added with continuous stirring to obtain a homogeneous mixture. The mixture was poured into a Teflon Petri dish (14 cm in diameter), allowed to dry, and pulled off from the Petri dish (Scheme 1). The thickness of the resulting film was measured by using a digital caliper with absolute technology, which was ∼ 0.09 mm.

#### Characterizations

Fourier Transforms Infrared Spectroscopy–Attenuated Total Reflectance (FTIR–ATR) The ATR spectra of CTA, CTA/ CaCO_3_/GO, and CTA/ CaCO_3_/GO/dye were recorded in the range of 500–4000 cm^− 1^ on a JASCO (Tokyo, Japan) FT-IR Spectrophotometer. The XRD patterns were investigated on a Diano X-ray diffractometer using a CuKα radiation source energized at 45 kV and a Philips X-ray diffractometer (PW 1930 generator, PW 1820 goniometer) with CuK radiation source (λ = 0.15418 nm), at a diffraction angle range of 2θ from 10 to 80° in reflection mode. The surface morphology and elemental composition of the films were studied using scanning electron microscopy (SEM) (Ta-scan (Vega3), Czech Republic) equipped with a non-destructive energy-dispersive X-ray (EDX) unit at an accelerating voltage of 30 kV. The UV–visible-NIR absorption spectrum was estimated in the 200–2300 nm range using a Spectrophotometer JASCO (V-570). Hydrophobic measurements, such as water-contacting angles of the fabricated films, were determined under the ASTM (D-7334) standard test using Dataphysics OCA15EC (Germany). The specific surface area and pore size of the films were evaluated using a specific surface area and porosity analyzer (Micromeritics of USA, TriStar II Plus Series).

#### Photocatalytic activity of CTA/ CaCO_3_/GO Film

In the first, the MB dye-removal efficiency was studied in the dark and under sunlight to elucidate the effect of sunlight on the photocatalytic removal of MB from water.

In the dark, the photocatalytic study was conducted at room temperature in a batch reactor. 50 mL of MB dye solution (100 mg/L) was prepared in distilled water, and 0.2 g of film was added with continuous stirring. a slurry composed of a dye solution and a film suspension was stirred magnetically and kept in the dark to establish adsorption–desorption equilibrium. The solution was withdrawn at different time intervals, and the absorbance was recorded (300–750 nm) by AELAB Double Beam UV–VIS Spectrophotometer (L7). The removal efficiency (R%) and the MB dye uptakes (q_e_) were calculated using the following equations:

1 $${\mathrm{R}}\% = \frac{{\left( {{C_o} - {C_t}} \right)}}{{{C_o}}}\; \times \;100$$

2 $${{\mathrm{q}}_{\mathrm{e}}} = \frac{{\left( {{C_o} - {C_t}} \right)}}{{\mathrm{M}}}\; \times \;{\mathrm{V}}$$

where *C*_*0*_ and *C*_*t*_ are the concentration of MB dye at the initial and time t. V is the volume of solution (mL), and M is the film mass (g).

In photocatalytic studies, to study the effect of sunlight on the photocatalytic removal of MB, the dye solution and film suspension were stirred for 15 min and then exposed to natural sunlight. The solution was withdrawn at different time intervals; the absorbance was recorded (300–750 nm) by AELAB Double Beam UV–VIS Spectrophotometer (L7), and R% and q_e_ were calculated. The structure of the MB dye is shown below:


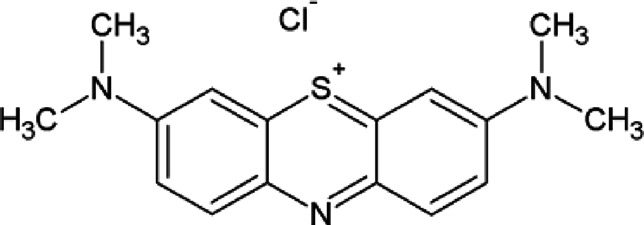


To investigate the effect of pH on removal efficiency, a standard batch equilibrium experiment was conducted. The pH of the MB dye solution was adjusted to 2–12 using NaOH and HCl. The other parameters were 0.2 g of film weight, 50 mL of MB dye solution, 2 h, and room temperature. To investigate the sorbent dosage effect, different weights (0.1, 0.15, and 0.2 g) of the film were added, ranging from 0.05 to 0.2 g. At the same time, other parameters were pH∼7, 50 mL MB dye solution, 2 h, and at room temperature.

#### Adsorption kinetics

Removal kinetics of MB dye from aqueous solution were illustrated by pseudo-first-order and pseudo-second-order.

3 $$\log \left( {{q_e} - {q_t}} \right) = \log \left( {{q_e}} \right) - \frac{{{K_1}}}{{22.303}}t$$

4 $$\frac{t}{{{q_e}}} = \frac{t}{{{q_e}}} + \frac{1}{{{K_2}q{e_2}\;\;}}$$

where qt and qe are the removal capacities of MB dye (mg/g) at time t and equilibrium, respectively. k1 is the rate constant of pseudo-first-order adsorption (min^− 1^). k2 is the rate constant of the pseudo-second-order sorption (g/mg min). The t/qt versus t plot was linear. The values of the adsorption parameters qe and k2 were determined from the slope and intercept of the Plot, respectively, where kp is the intraparticle diffusion rate constant.

## Results and discussion

### Preparation and characterization of modifying components

**Fig. 1:**
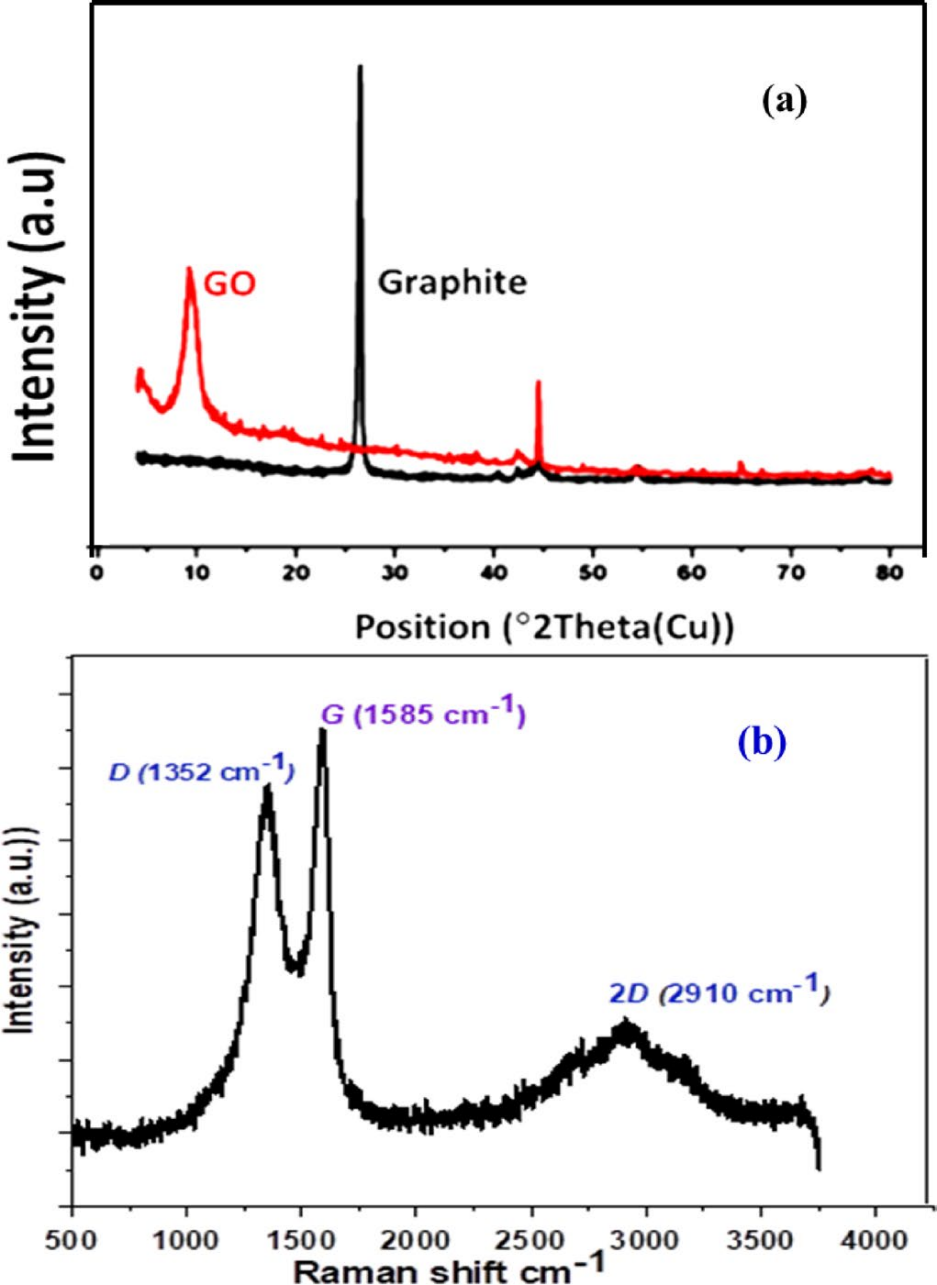
XRD pattern of graphite (**a**) and GO, and Raman spectra of GO (**b**).

Graphite was oxidized to GO using a modified Hummers’ oxidation method; the oxidation was confirmed by X-ray diffraction analysis. Figure 1a shows a sharp peak at 2θ ~ 27° in the case of graphite, while GO shows an intense diffraction ray at 2θ ~ 10°. The disappearance of the diffraction at 2–27 confirms the introduction of oxygen-containing functionalities, such as epoxy, hydroxyl, and carboxyl groups, and the success of the oxidation process^29^. To confirm graphite oxidation, the oxidized graphite was analyzed by Raman spectroscopy (Fig. 1b). It can be seen from Fig. 1b that there are three prominent characteristic peaks^30^; the D peak at 1355 cm^− 1^ arising from the doubly resonant disorder-induced mode due to the stretching of C–C bond; the G peak at 1585 cm^− 1^ due to the first-order scattering of the E^2^ g phonon of the sp^2^ C at the Brillouin zone center and 2D peak at 2910 cm^-1^ due to the existence of oxygenated molecules that helped to prevent the graphene layers from stacking^31^.

**Fig. 2:**
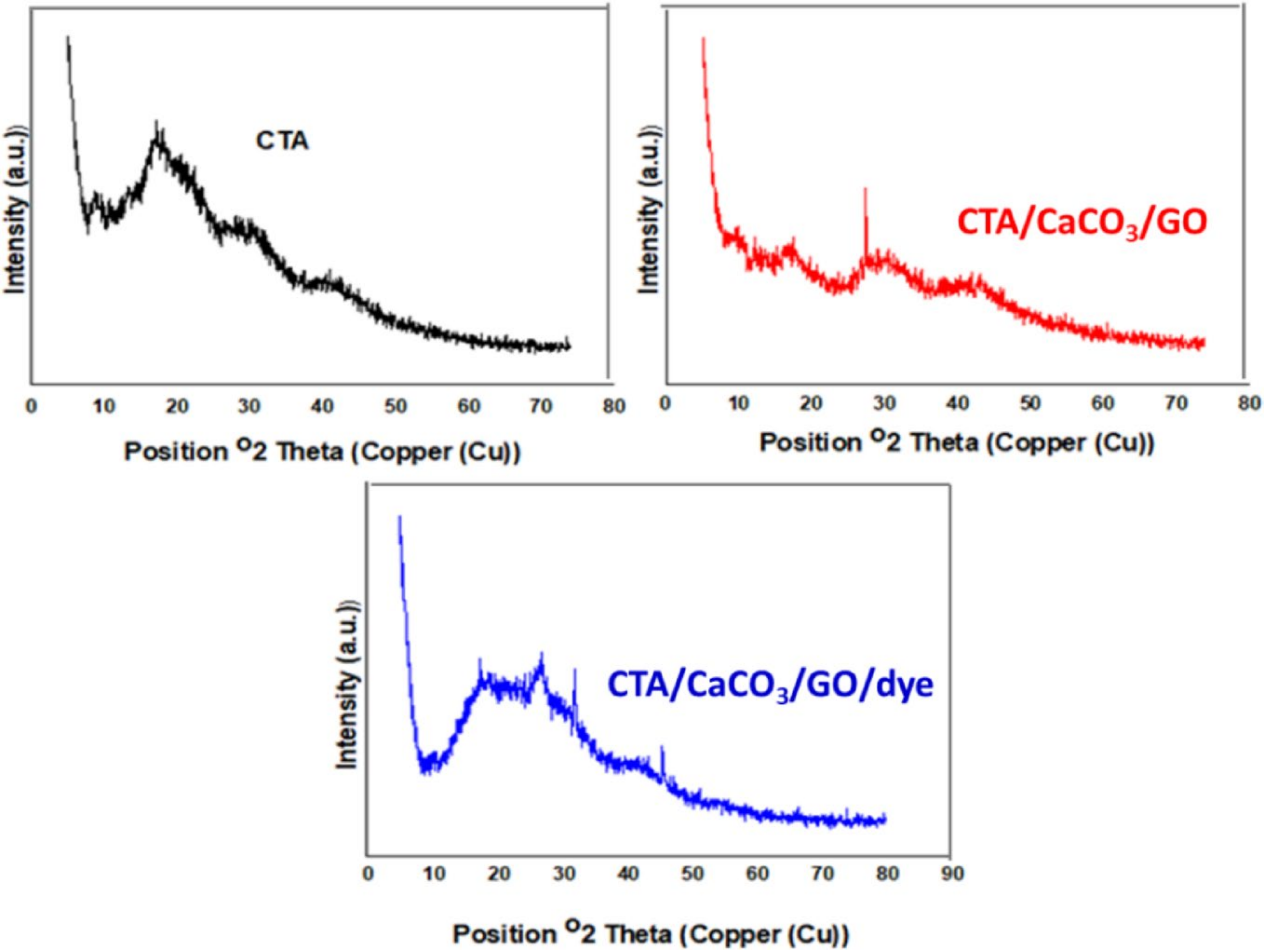
XRD pattern of CTA, CTA/CaCO_3_/GO, and CTA/CaCO_3_/GO/dye films.

Since the XRD evaluations can be used to examine the structural organization of materials, the XRD diffractograms of CTA were used to investigate the combination of CTA with GO. Figure 2 shows the XRD patterns of CTA, CTA/ CaCO_3_/GO, and CTA/ CaCO_3_/GO/dye films. It is well established that CTA is composed of blocks of pyranose rings linked to acetyl groups^29^. As shown in Fig. 2, XRD patterns of CTA and CTA/CaCO₃/GO films exhibit the typical broad diffraction feature near 2θ ≈ 18°, commonly associated with amorphous or less-ordered domains in CTA, alongside weaker reflections at higher angles^30^. In the composite, the relative intensity of the 18° feature decreases, which we attribute to filler-induced constraints on segmental mobility and local packing rearrangements within the CTA matrix rather than a definitive increase in crystallinity^31,32^. The absence of a distinct GO peak suggests that exfoliated or well-dispersed GO sheets are below the detection threshold for layered stacking. Given that peak intensity alone cannot substantiate changes in crystallinity. Accordingly, we interpret the XRD changes as indicative of matrix reorganization and interfacial interactions with CaCO₃/GO, without claiming an increase in crystalline sites^33^. After the adsorption of MB dye by CTA/ CaCO_3_/GO film, the XRD pattern was slightly changed as the peaks at 18° and 30° overlapped. The diffraction peaks at 28, 30, and 46° can be assigned to CaCO_3_. These peaks revealed that CaCO_3_ is trapped inside CTA^32^. The diffraction peaks observed at 2θ ≈ 28°, 30°, and 46° in the CTA/CaCO₃/GO film correspond to the crystalline phases of CaCO₃. These reflections can be indexed to calcite according to the standard JCPDS card No. 05-0586, which reports characteristic peaks at 2θ ≈ 29.4° (104), 39.4° (113), and 47.5° (202). The slight shifts in peak positions may be attributed to matrix interactions within the CTA film. Indexing with the JCPDS card no. 47-1713 confirms the successful incorporation of CaCO₃ into the composite structure^33^.

**Fig. 3:**
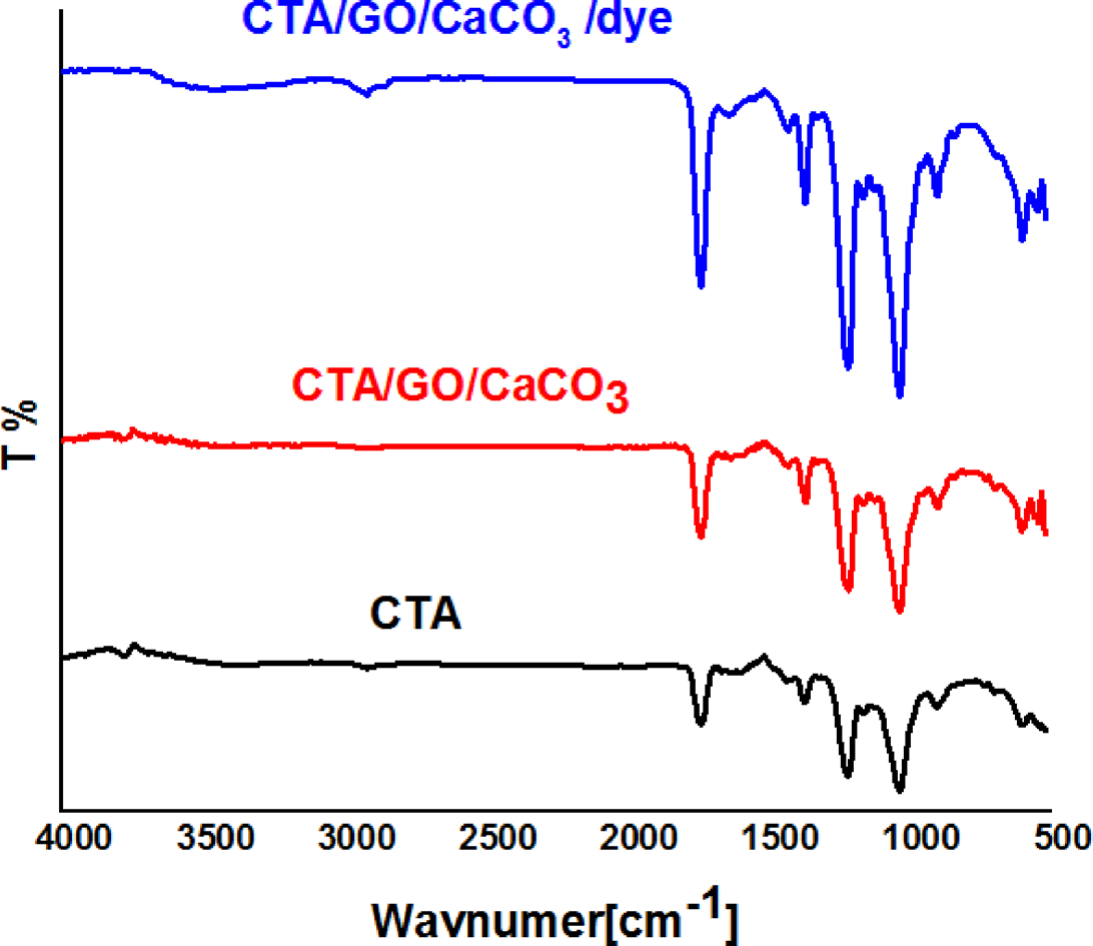
FTIR of CTA, CTA/CaCO_3_/GO, and CTA/CaCO_3_/GO/dye films.

The FTIR-ATR spectrum was used to identify the sample’s functional groups, thereby providing information on its chemical composition. Figure 3 shows the spectrum of the CTA, CTA/GO, and CTA/GO after the adsorption of dye. The absorption band around 1745 cm^− 1^ is attributed to stretching vibrations of the –C = O of CTA and GO, and the intensity of this band was increased with the loading of GO onto CTA. The increase in intensity after MB adsorption may be attributed to the –CH=N bond^34^. Bands at 1220 and 1040 cm^− 1^ correspond to the stretching modes of C–O single bonds. The bands at 1400 cm^− 1^ correspond to the stretching modes of C = N bonds of MB. A small OH peak in cellulose triacetate is a characteristic feature in the 3600–3150 cm^− 1^ region, attributed to stretching modes^35,36^. The appearance of a new band at 1040 cm^− 1^ in the spectra of CTA/CaCO_3_/GO can be assigned to doubly degenerate planar asymmetric stretching of CaCO_3_; the intensity of this band was increased with the loading of MB dye. Additionally, a clear band has appeared at 820, which is associated with out-of-plane bending^37–39^.

#### Surface morphology and EDX analyses

**Fig. 4:**
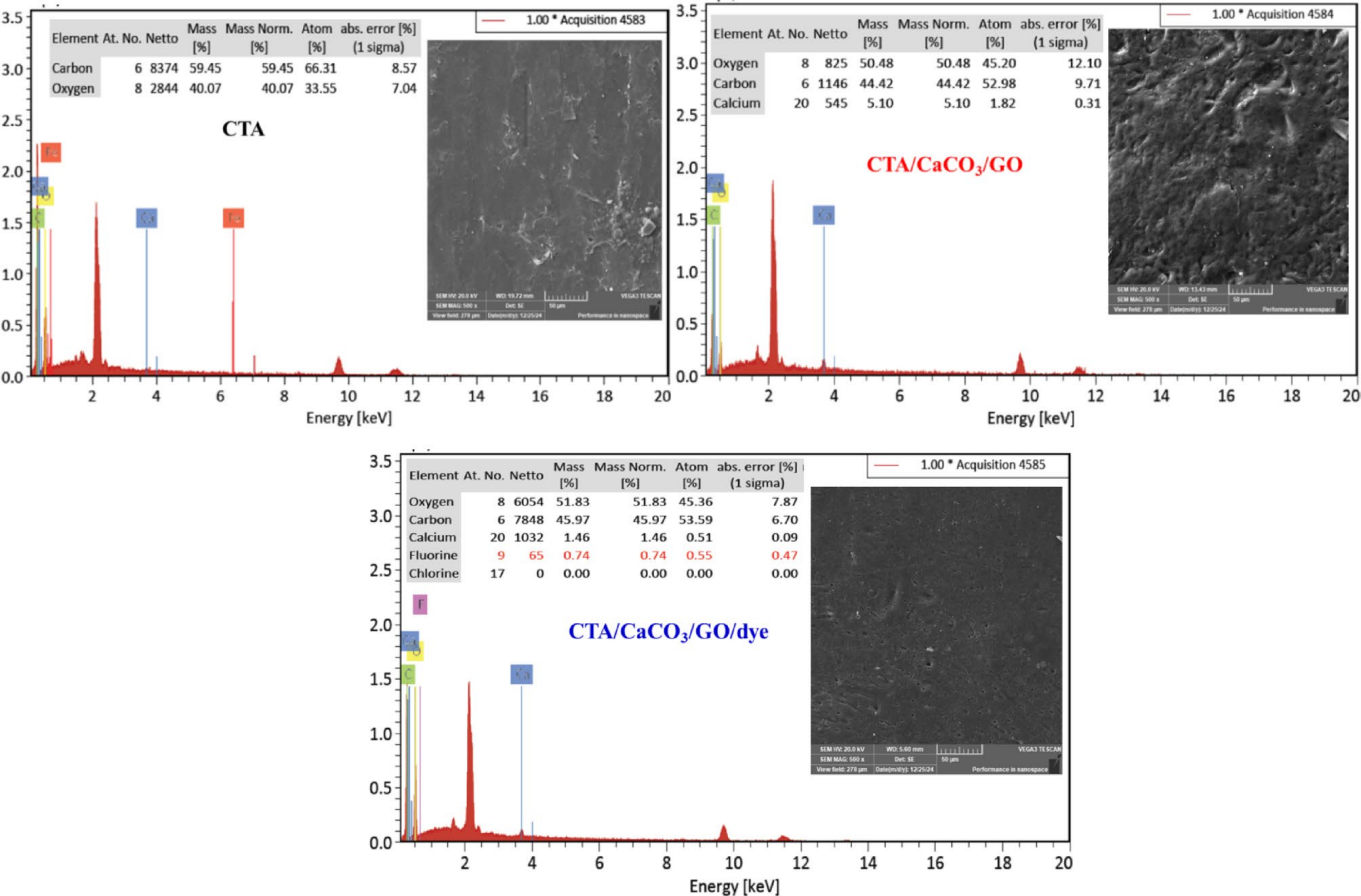
SEM images and EDX analysis of CTA, CTA/CaCO_3_/GO, and CTA/CaCO_3_/GO/dye films.

To evaluate the effect of loading with the additives CaCO_3_ and GO on the final structure of the prepared CTA films, the morphology of the most significant films was investigated using SEM. Figure 4 shows the SEM surface images of the films and EDX elemental analysis. It can be observed that pores are present in the CTA film, attributable to the volatility of dioxin/acetone, their evaporation during the drying process. Analysis of the images after incorporating the additives CaCO_3_ and GO revealed a porous surface with voids and cavities of various shapes and sizes, attributable to the reaction of CaCO_3_. After MB adsorption, the adsorbent film, CTA/CaCO_3_/GO/dye, exhibits a smooth, homogeneous surface, suggesting that the pores were filled with MB molecules. This conclusion is supported by the XRD and FTIR spectra analysis of CTA/CaCO_3_ and CTA/CaCO_3_/GO/dye films. The EDX spectra of CTA, CTA/CaCO_3_/GO, and CTA/CaCO_3_/GO/dye films indicate the presence of various elements, primarily C and O in CTA, and reveal oxygen-containing functional groups, as observed by FTIR. In the spectra of CTA/CaCO_3_/GO and CTA/CaCO_3_/GO/dye, the Ca peak appeared, indicating the loading of CaCO_3_. The presence of Cl peaks further supported the adsorption of MB on CTA/CaCO_3_/GO.

#### BET surface porosity

**Fig. 5:**
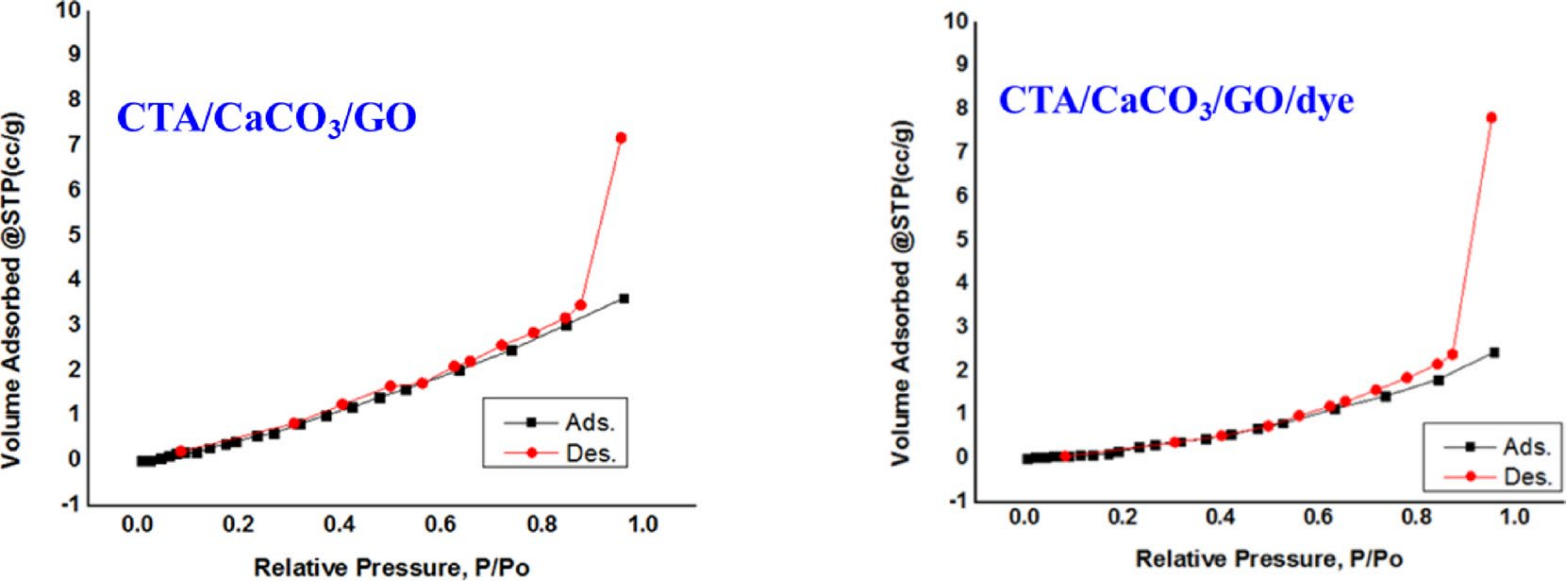
BET analysis CTA/CaCO_3_/GO and CTA/CaCO_3_/GO/dye films.

To understand the adsorption and photocatalyst properties of the CTA/CaCO₃/GO film, the surface physical properties, such as surface area, average pore radius, and total pore volume, were measured by nitrogen adsorption-desorption isotherm (Fig. 5). Figure 5 shows the nitrogen physisorption isotherm of the film before and after MB treatment. Physisorption is characterized by Type II and Type IV isotherms, with reversible adsorption and desorption resulting from monolayer and multilayer formation, which are characteristic of macroporous and mesoporous adsorbents. The CTA/CaCO₃/GO film showed a surface area of 6.46 m²/g, an average pore radius of 33.01 nm, and a pore volume of 5.33 cm³/g. After dye incorporation (CTA/CaCO₃/GO/dye), the surface area decreased to 1.20 m²/g, and both pore radius and pore volume were reduced to 14.53 nm and 4.37 cm³/g, respectively. This reduction suggests that MB molecules occupied and partially blocked the pores, reducing access to active sites. This pore-filling behavior aligns with adsorption-driven dye uptake prior to photodegradation. Following MB adsorption, MB molecules are more efficiently degraded under solar irradiation because GO enhances charge separation and light absorption. This demonstrates the film’s dual function as both an adsorbent and a photocatalyst, which enhances wastewater treatment by enabling effective capture and degradation of pollutants^40^ (Table 1).

**Table 1:** Surface area (m²/g), average pore radius (nm), and total pore volume (cm3/g) of CTA, CTA/CaCO_3_/GO, and CTA/CaCO_3_/GO/dye films.

Film	Surface area (m²/g)	Average pore radius (nm)	Total pore volume (cm^3^/g)
CTA/CaCO_3_/GO	6.4613	33.012	5.332
CTA/CaCO_3_/GO/dye	1.2045	14.529	4.375

#### UV–Vis spectroscopy

**Fig. 6:**
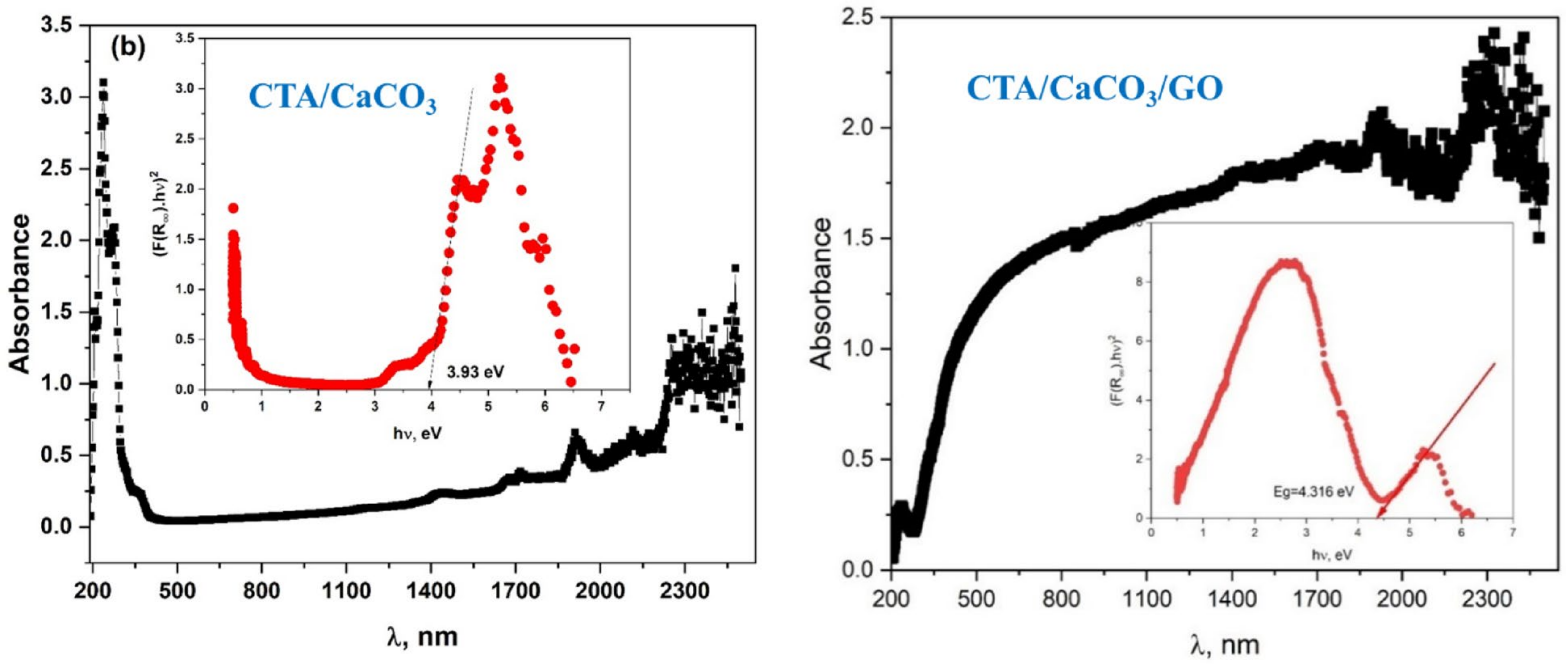
Absorption spectra and Tauc’s plot of CTA/CaCO_3_ and CTA/CaCO_3_/GO films.

The optical analysis was conducted using an ultraviolet-visible spectrophotometer to determine the absorption rate and band gap energy. Figure 6 displays the optical absorbance spectrum of CTA/CaCO_3_ and CTA/CaCO_3_/GO films, where absorbance is plotted as a function of wavelength (λ) in nanometers (nm).

The spectrum of the CTA/CaCO₃ film shows strong absorbance in the ultraviolet region (200–400 nm), exceeding 3.0 units, which is attributed to π→π* and n→π* transitions of carbonyl and ester groups in CTA. Additionally, the film exhibits minimal absorption across the visible range (400–700 nm), confirming its transparency^41,42^. The Tauc plot analysis yields a direct band gap of ~ 3.93 eV, consistent with insulating behavior and strong UV-blocking capacity. Overall, the CTA/CaCO₃ film integrates high UV absorbance, visible transparency, and a wide band gap. On the other hand, the absorbance curve of CTA/CaCO_3_/GO film gradually increases from around 200 nm, reaching a plateau in the near-infrared region. This trend suggests that CTA/CaCO_3_/GO film has significant absorption in the UV–VISible range, with increasing absorbance at shorter wavelengths, indicating electronic transitions within the material^43,44^. The inset in Fig. 6 shows the Tauc plot for an indirect bandgap transition, used to estimate the optical bandgap energy (Eg). The Tauc plot represents (F(R_∞_)hν)^2^ as a function of photon energy (hν), where F(R_∞_) is the Kubelka-Munk function derived from diffuse reflectance data. Extrapolating the linear region of the plot to the energy axis yields the material’s optical bandgap^45^. From the inset, the estimated bandgap energy of CTA/CaCO_3_/GO film is 4.316 eV, suggesting that the material is a wide-bandgap semiconductor. This wide bandgap value indicates that CTA/CaCO_3_/GO film could be suitable for applications in optoelectronic devices, such as UV photodetectors, transparent conductive films, or photocatalytic materials^46–48^. Broad absorption in the visible and near-infrared ranges may also indicate potential for solar energy harvesting or luminescent applications^49^. The absorption features and the nature of the bandgap transition provide insights into the material’s electronic structure and potential technological applications.

The CTA/CaCO₃ film exhibits strong UV absorbance and a band gap of 3.93 eV, which facilitates rapid electron–hole generation and direct oxidative degradation of dye molecules under high-energy UV irradiation. Nevertheless, its photocatalytic activity remains largely confined to the UV region, thereby reducing its efficiency under natural sunlight, where visible light is predominant. In contrast, the CTA/CaCO₃/GO film, although exhibiting a wider band gap of 4.316 eV, is due to the synergistic effects of GO. GO promotes charge separation, inhibits electron–hole recombination, and offers numerous adsorption sites for dye molecules through π–π interactions. These characteristics extend photocatalytic activity into the visible region and enhance stability during prolonged irradiation^50^. Although the CTA/CaCO₃ film demonstrates strong intrinsic UV activity, the CTA/CaCO₃/GO film offers greater advantages for wastewater treatment, where both combined UV–Vis irradiation and long-term operational stability are essential. The incorporation of GO enhances photocatalytic efficiency and increases pollutant adsorption, establishing CTA/CaCO₃/GO as a more sustainable approach for dye photodegradation in water remediation. Consequently, the CTA/CaCO₃/GO film was selected for the photodegradation of MB.

#### Hydrophobic characterization

**Fig. 7:**
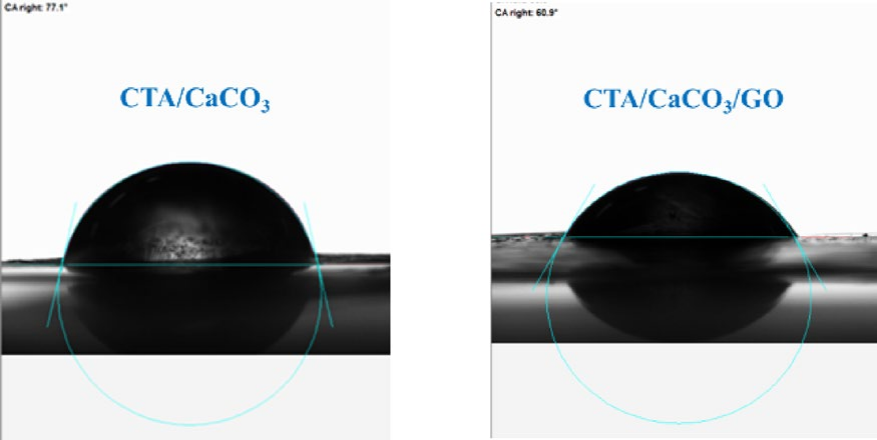
Contact angle images of CTA/CaCO_3_ and CTA/CaCO_3_/GO films.

Figure 7 illustrates the water contact angle of the CTA/CaCO₃ and CTA/CaCO₃/GO films, providing insight into the hydrophilic behavior of the prepared films. The contact angle of the CTA/CaCO₃ film was 77.1°, indicating that the CTA/CaCO₃ film has a moderately hydrophobic surface. This behavior is attributable to CaCO₃, which may increase surface roughness and partially shield the CTA’s polar functional groups, limiting their direct interaction with H_2_O. The incorporation of GO into CTA/CaCO₃ slightly decreased the contact angle (60.9º), indicating a significant increase in hydrophilicity. This increase in hydrophilicity is attributable to oxygen-containing functional groups on GO sheets, such as –OH, –COOH, and epoxy groups, which promote strong hydrogen bonding with H_2_O^51^. Additionally, the uniform dispersion of GO within the CTA can facilitate exposure of the hydrophilic groups at the film surface, enhancing the hydrophilicity. This decrease in contact angle indicates that GO’s hydrophilic properties outweigh the potential hydrophobic or roughness effects of CaCO₃, confirming GO’s effectiveness as a surface-modifying agent and thereby transforming the CTA/CaCO₃ composite into a more hydrophilic material.

#### Photocatalytic activity of CTA/CaCO_3_/GO film

In general, the photocatalytic activity of the adsorbent is the photodegradation of molecules under solar irradiation, driven by reactive species formed during irradiation that mediate oxidative degradation into smaller products. While the adsorption is the physical/chemical binding of molecules onto the surface functional groups of the adsorbent, this process can occur in the dark and establishes an adsorption–desorption equilibrium. In this work, the solar photocatalytic activity of the CTA/CaCO_3_/GO film was evaluated for the removal of MB at room temperature over 2 h, using 0.2 g of film. For adsorption equilibrium experiments conducted in the dark, a relatively high MB concentration (100 mg/L) was used to ensure measurable uptake and better evaluate the adsorption capacity of the CTA/CaCO₃/GO film under non-irradiated conditions. In contrast, for solar photodegradation experiments, a lower MB concentration (0.05–0.2 mg/L) was used to simulate environmentally relevant dye levels typically in wastewater and to facilitate efficient degradation under natural sunlight. This dual approach allowed for a comprehensive assessment of both adsorption behavior at elevated pollutant loads and photocatalytic performance under realistic wastewater conditions. It was observed that MB removal was higher under solar light than in the dark, confirming greater MB degradation by the film. The high degradation of MB on CTA/CaCO_3_/GO was attributed to the presence of both CaCO_3_ and GO in the composite film. Moreover, the high solar photodegradation activity of CTA/CaCO_3_/GO may be due to the simultaneous adsorption and photocatalytic activity of the film materials^52^. The mechanism of solar photocatalytic degradation of MB onto CTA/CaCO_3_/GO is shown below. Under solar irradiation, electrons from the conduction band were transferred to the catalyst surface, producing an electron–hole pair. The O_2_ was reduced to the OH radical by electrons at the conduction band. At the same time, OH–/H_2_O formed an OH radical by reacting with the valence band holes^53^. The OH radicals were responsible for the MB degradation, and the plausible mechanism is:

$${\mathrm{CTA/CaC}}{{\mathrm{O}}_3}/{\mathrm{GO}} + {\mathrm{MB}} \to {\mathrm{CTA/CaC}}{{\mathrm{O}}_3}{\mathrm{/GO-MB}}_{{\mathrm{ads}}}$$

$${\mathrm{CTA/CaC}}{{\mathrm{O}}_3}{\mathrm{/GO-M}}{{\mathrm{B}}_{{\mathrm{ads}}}} + {\text{ h}}\nu \to {\mathrm{CTA/CaC}}{{\mathrm{O}}_3}{\mathrm{/GO-M}}{{\mathrm{B}}_{{\mathrm{ads}}}}{*_{{\mathrm{ads}}}}$$

$${\mathrm{CTA/CaC}}{{\mathrm{O}}_3}{\mathrm{/GO-M}}{{\mathrm{B}}_{{\mathrm{ads}}}}{*_{{\mathrm{ads}}}} \to {{\mathrm{e}}^ - }{\mathrm{CB}} + {{\mathrm{h}}^ + }_{{\mathrm{VB}}}$$

$${{\mathrm{h}}^ + } + {{\mathrm{H}}_2}{\mathrm{O}} \to {\mathrm{H}}{{\mathrm{O}}^ \cdot } + {{\mathrm{H}}^ + }$$

$${{\mathrm{O}}_2} + {\text{ }}{{\mathrm{e}}^ - } \to {{\mathrm{O}}_2}^ -$$

$${{\mathrm{O}}_2}^ - + {\text{ }}{{\mathrm{H}}^ + } \to {\mathrm{H}}{{\mathrm{O}}_2}$$

$$2{\mathrm{H}}{{\mathrm{O}}_2} \to {{\mathrm{H}}_2}{{\mathrm{O}}_2} + {{\mathrm{O}}_2}$$

$${{\mathrm{H}}_2}{{\mathrm{O}}_2} + {\text{ }}{{\mathrm{O}}_2}^ - \to 2{\mathrm{OH}} + {{\mathrm{O}}_2}$$

$${\mathrm{OH+MB}}\to{\mathrm{Degraded}\;\mathrm{product}}$$

In the first, we studied the removal of MB dye from water using a CTA/CaCO_3_/GO film in the absence of solar irradiation and under solar irradiation. Approximately 14% of MB was removed in 2 h, compared with 90% under the same conditions with solar radiation exposure. This can be explained by the adsorption of MB molecules onto available surface functional groups (–OH, –COOH, and Ca²⁺ sites) in the dark. The increase in the removal efficiency under solar irradiation indicates that photocatalytic degradation played a dominant role beyond simple adsorption. Adsorption is the initial uptake of MB molecules onto the film surface, establishing adsorption–desorption equilibrium, whereas photocatalytic degradation involves the subsequent oxidative degradation of the adsorbed MB. Thus, the CTA/GO/CaCO₃ film operates via a dual mechanism: adsorption concentrates MB at the film surface, and photocatalysis degrades MB into smaller, colorless products under solar light. These results are consistent with the kinetic analysis in the coming section. Consequently, we further investigate the effects of pH, contact time, and sorbent dosage on the removal efficiency of MB from water.

#### Effect of pH

**Fig. 8:**
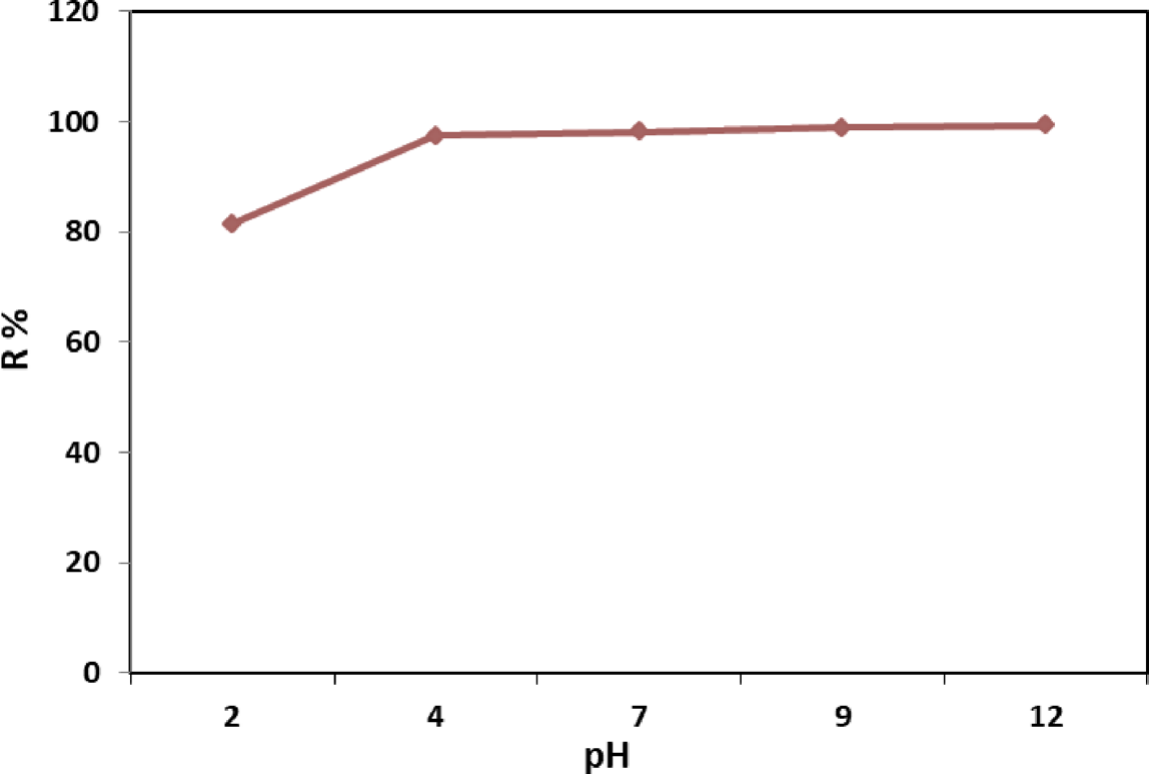
Effect of pH solution on the MB removal efficiency by CTA/CaCO_3_/GO film (0.2 g) after 2 h and MB (25 mg/L).

pH is one of the most significant factors influencing the photocatalytic degradation of wastewater, as it affects the charge on aggregates and catalyst particles, and the positions of the conduction and valence bands, thereby influencing the adsorption of dyes at the film surface. Consequently, the photocatalytic activity of CTA/CaCO_3_/GO film was determined for the degradation of MB at various pH. The pH value significantly impacts the removal efficiency of contaminants from wastewater. Figure 8 represents the removal capacity of MB by 0.2 g of CTA/CaCO_3_/GO at a pH range (2–12) and exposed to natural solar light radiation. The data showed that the removal efficiency increased with increasing pH. Less MB removal is noticed at low pH values due to the presence of a large number of (H^+^) protons that may compete with MB to chelate to the CTA/CaCO_3_/GO sites. At high pH, H + was absent, and the negative charge increased attraction to MB, thereby increasing removal^54^. The results demonstrate that the optimal pH for MB removal is 4 (removal efficiency ~ 100%). This efficiency remains nearly constant from pH 4–12, indicating the stability of the CTA/CaCO3/GO film across a wide pH range, which can be attributed to strong electrostatic attraction between the cationic MB and the negatively charged film surface at pH ≥ 4, the buffering role of CaCO₃ which minimizes structural changes under alkaline conditions, and the resilience of both GO functional groups and the CTA polymer backbone that preserve adsorption sites. Consequently, the CTA/CaCO₃/GO film exhibits high efficiency and broad pH tolerance, making it suitable for wastewater treatment across a wide pH range.

#### Effect of sorbent dosage

**Fig. 9:**
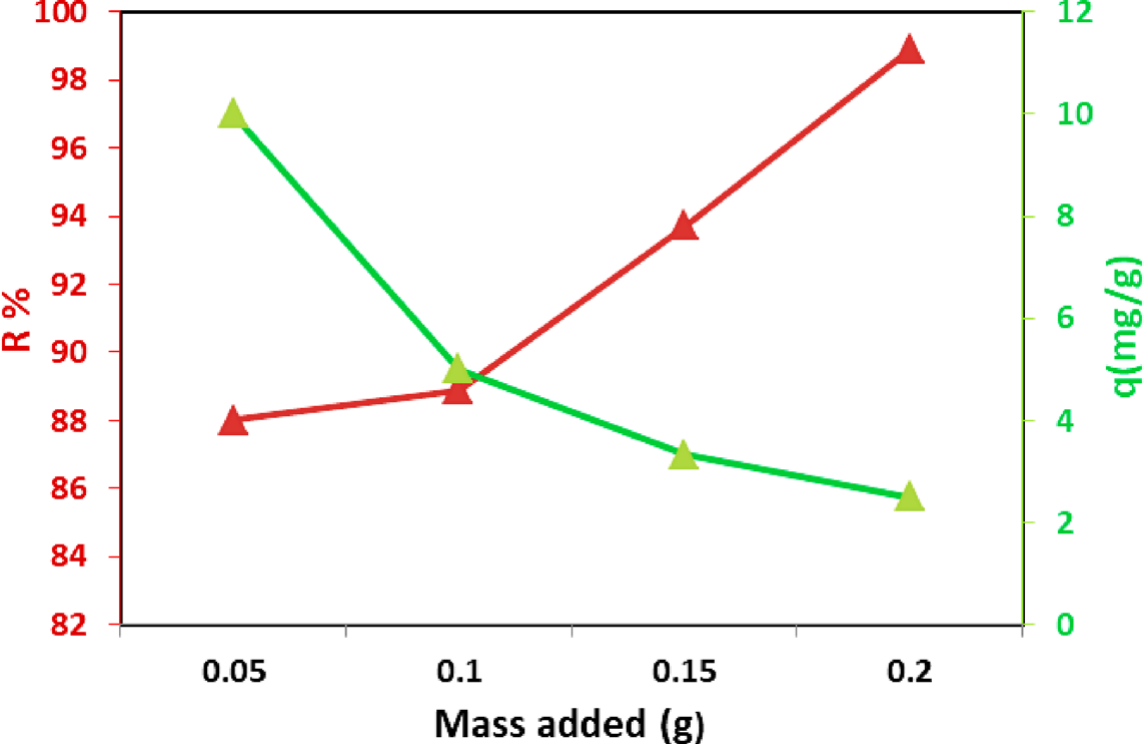
Effect of MB dosage on the removal efficiency by CTA/CaCO_3_/GO film (0.2 g) after 2 h, at pH∼7, and MB (0.05–0.2 mg/L).

The sorbent dosage used to remove MB from water is a critical factor. Figure 9 shows the effect of CTA/CaCO_3_/GO film doses (0.05–0.2 g) on the removal efficiency (R) and the uptake capacity (q), with other parameters (time, pH, temperature, and dye concentration) held constant, and under natural sunlight. As the film dose increased, removal efficiency increased due to an increase in the number of surface sites available for MB adsorption. The adsorption capacity decreased with increasing dose. This may be due to the increasing number of active groups on the surface sites at higher doses, which enhances removal efficiency.

#### Effect of contact time

**Fig. 10:**
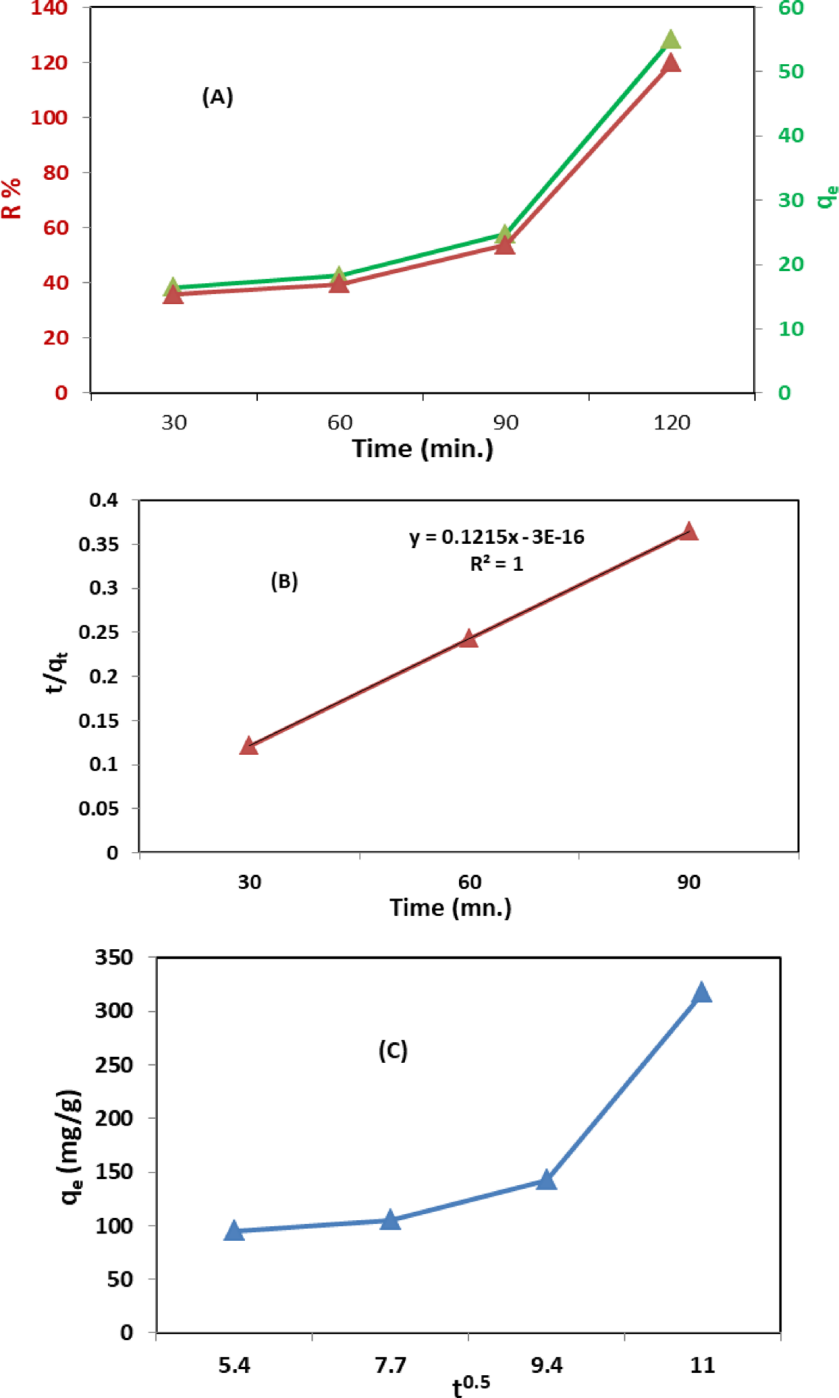
Effect of time on the MB removal efficiency by CTA/CaCO_3_/GO film (**A**), the pseudo-second-order (**B**), and the Intraparticle diffusion model (**C**).

The contact time between the adsorbent and the MB dye is crucial for determining removal efficiency. To determine the time required for optimal removal efficiency, experiments were conducted using 0.2 g of CTA/CaCO_3_/GO film in a 50 mg/L MB dye solution for 2 h at room temperature, and the film was exposed to natural sunlight (Fig. 10A).

The data show that removal efficiency increases with time, reaching 90% at 120 min, owing to sufficient active groups, such as COOH, OH, and Ca, on the surface. These active sites became occupied with MB dye. The kinetic studies of MB dye removal by CTA/CaCO_3_/GO were analyzed using the pseudo-second-order model (Fig. 10B) and the intra-particle diffusion model (Fig. 10C), and the correlation coefficient for the pseudo-second-order model was 1.0. Thus, it can be concluded that both adsorption and photocatalysis may contribute to MB removal, and that chemisorption may play a role. The linear plot confirmed that MB adsorption onto the CTA/CaCO₃/GO film follows the pseudo-second-order kinetic model. This model implies that the rate-limiting step involves electron sharing or exchange between the adsorbent and the adsorbate, a characteristic of chemisorption. The presence of functional groups such as –OH and –COOH on GO and CTA, along with Ca²⁺ from CaCO₃, facilitates strong chemical interactions with MB molecules, including electrostatic attraction, hydrogen bonding, and π–π stacking. These findings support the conclusion that MB removal is governed by chemisorption and follows a pseudo-second-order rate law^55^. Physicoadsorption was slight, which may be due to the nature of the CTA surface, which doesn’t permit physicoadsorption. Therefore, the use of CaCO_3_ to reduce the compacted nature of CTA and increase the number of pores on its surface is preferred.

## Conclusion

- The preparation of a CTA/CaCO_3_/GO film was investigated for the solar photodegradation of MB in wastewater.
- The contact angle results demonstrate that the synergistic incorporation of CaCO₃ and GO enables tunable surface properties, thereby facilitating the tailoring of CTA-based films for water-related applications.
- The FTIR and XRD spectra confirmed the formation of the composite film. The bandgap values of the CTA/CaCO_3_/GO film confirm its semiconducting nature.
- BET analysis indicates that the film serves dual functions as an adsorbent and a photocatalyst, thereby improving wastewater treatment by efficiently capturing and degrading pollutants.
- CTA/CaCO_3_/GO exhibited high ion-exchange capacity and significant MB photodegradation, and was highly efficient for solar photodegradation.
- The CTA/CaCO₃/GO film exhibits high efficiency and broad pH tolerance, making it suitable for wastewater treatment across a wide pH range.
- The kinetic study of the solar photodegradation of MB dye in the CTA/CaCO_3_/GO film followed a pseudo-second-order model.

## Data Availability

The data presented in this study are openly available in the article.
